# Effectiveness of an Innovative Cognitive Treatment and Telerehabilitation on Subjects With Mild Cognitive Impairment: A Multicenter, Randomized, Active-Controlled Study

**DOI:** 10.3389/fnagi.2020.585988

**Published:** 2020-11-16

**Authors:** Rosa Manenti, Elena Gobbi, Francesca Baglio, Ambra Macis, Clarissa Ferrari, Ilaria Pagnoni, Federica Rossetto, Sonia Di Tella, Federica Alemanno, Vincenzo Cimino, Giuliano Binetti, Sandro Iannaccone, Placido Bramanti, Stefano F. Cappa, Maria Cotelli

**Affiliations:** ^1^Neuropsychology Unit, IRCCS Istituto Centro San Giovanni di Dio Fatebenefratelli, Brescia, Italy; ^2^IRCCS, Fondazione Don Carlo Gnocchi – ONLUS, Milan, Italy; ^3^Service of Statistics, IRCCS Istituto Centro San Giovanni di Dio Fatebenefratelli, Brescia, Italy; ^4^Department of Rehabilitation and Functional Recovery, IRCCS San Raffaele Hospital and Scientific Institute, Vita-Salute San Raffaele University, Milan, Italy; ^5^IRCCS Centro Neurolesi “Bonino Pulejo,” Messina, Italy; ^6^MAC Memory Clinic and Molecular Markers Laboratory, IRCCS Istituto Centro San Giovanni di Dio Fatebenefratelli, Brescia, Italy; ^7^NEtS, Scuola Universitaria Superiore IUSS-Pavia, Pavia, Italy; ^8^IRCCS Fondazione Mondino, Pavia, Italy

**Keywords:** cognitive, telerehabilitation, dementia, mild cognitive impairment, home

## Abstract

**Background:**

In recent years, the potential usefulness of cognitive training procedures in normal aging and mild cognitive impairment (MCI) have received increased attention.

**Objective:**

The main aim of this study was to evaluate the efficacy of the face-to-face cognitive virtual reality rehabilitation system (VRRS) and to compare it to that of face-to-face cognitive treatment as usual for individuals with MCI. Moreover, we assessed the possibility of prolonging the effects of treatment with a telerehabilitation system.

**Methods:**

A total of 49 subjects with MCI were assigned to 1 of 3 study groups in a randomized controlled trial design: (a) those who received face-to-face cognitive VRRS (12 sessions of individualized cognitive rehabilitation over 4 weeks) followed by telerehabilitation (36 sessions of home-based cognitive VRRS training, three sessions for week); (b) those who received face-to-face cognitive VRRS followed by at-home unstructured cognitive stimulation (36 sessions of home-based unstructured cognitive stimulation, three sessions for week); and (c) those who received face-to-face cognitive treatment as usual (12 sessions of face-to-face cognitive treatment as usual).

**Results:**

An improvement in memory, language and visuo-constructional abilities was observed after the end of face-to-face VRRS treatment compared to face-to-face treatment as usual. The application of home-based cognitive VRRS telerehabilitation seems to induce more maintenance of the obtained gains than home-based unstructured stimulation.

**Discussion:**

The present study provides preliminary evidence in support of individualized VRRS treatment and telerehabilitation delivery for cognitive rehabilitation and should pave the way for future studies aiming at identifying optimal cognitive treatment protocols in subjects with MCI.

**Clinical Trial Registration:**

www.ClinicalTrials.gov, identifier NCT03486704.

## Introduction

In recent years, there has been growing interest in the use of telerehabilitation methods in patients with neurodegenerative diseases (Cherney and van Vuuren, 2012; Cotelli et al., 2019).

Given the limited effectiveness of pharmacological treatments, there is a critical need to develop novel interventions aimed at preventing or delaying the onset of Alzheimer’s disease (AD), and mild cognitive impairment (MCI) might represent a potential target for intervention trials (Kidd, 2008; Hong et al., 2015; Janoutova et al., 2015).

Traditional cognitive training involves intensive in-person sessions that may not prove to be feasible and cost-effective in the case of large-scale implementation. The average lifespan in the world almost doubled during the 20th century and has resulted in a large number of people living to old ages, causing an increased risk of developing age-related diseases, disability and dementia (Fratiglioni et al., 1999; Brown, 2015). In the coming years, the growing demand and the need to contain the costs of health care will dictate the need to reorganize the services dedicated to people at risk of developing cognitive impairment by taking advantage of technological developments (Bharucha et al., 2009; Astell, 2019; Moyle, 2019). Telerehabilitation via information and communication technologies (Brennan et al., 2011; Realdon et al., 2016; Pitt et al., 2019) represents an innovative approach to overcome the obstacles associated with face-to-face intervention. Telerehabilitation technologies allow to provide services remotely in patients’ homes or other environments, allowing access to health care to patients living in rural settings or with mobility difficulties (Brennan et al., 2002, 2009, 2011; Forducey et al., 2003; Mashima and Doarn, 2008; Zampolini et al., 2008; Hailey et al., 2011; Peretti et al., 2017). In addition, the telerehabilitation modality offers the advantage of providing rehabilitation within the natural environment of the patient’s home, making the treatment more realistic and possibly more generalizable to the person’s daily life (McCue et al., 2010).

Recent studies have shown that the application of telerehabilitation methodology in individuals with physical impairments, post-stroke participants and patients with neurodegenerative diseases leads to clinical improvements that are generally equal to those induced by conventional face-to-face rehabilitation programs (Brennan et al., 2002; Rosen, 2004; Poon et al., 2005; Mashima and Doarn, 2008; Kairy et al., 2009; Cherney and van Vuuren, 2012; Jelcic et al., 2014; Antonietti et al., 2016; Vermeij et al., 2016; Burton and O’Connell, 2018; Isernia et al., 2019).

A recent systematic review showed the efficacy of telerehabilitation on cognitive abilities in individuals with MCI and in patients with neurodegenerative diseases associated with cognitive impairment (Cotelli et al., 2019). MCI is a condition associated with risk of progression to dementia, and represents a well-suited target for prevention studies (Petersen et al., 1999, 2014; Petersen, 2004; Livingston et al., 2017). However, these treatments are delivered in several ways and there is not a clinical consensus about content-design of telerehabilitation. A fixed schedule approach has proved to be effective in the treatment of elderly people with high risk of conversion in dementia resulting in a significant improvement in global cognitive functioning, memory and processing speed (Lampit et al., 2014). In other studies, participants were given the opportunity to choose free among the activities available in each session of training (Medalia and Freilich, 2008; Gooding et al., 2016). A third alternative consisted in the user-centered approach, which customized the choice of rehabilitative contents based on the performance obtained by the individual to the set up tests implemented in the software at the beginning of the rehabilitation path (Solana et al., 2015; Vance et al., 2018). So far, the majority of studies are feasibility or pilot studies with small-medium sample size and are very heterogeneous in terms of intensity and duration of treatment (Burdea et al., 2015; Espay et al., 2016; Dodakian et al., 2017). For this reason, in the light of this ongoing deep transformation of health care, it is of great relevance the effort to harmonize intervention protocols and randomized controlled trials (RCTs) are strongly needed to demonstrate the effectiveness of these home-based technology-enhanced treatment protocols with respect to the gold-standard, named the usual face-to-face care (Linden et al., 2016; Fetta et al., 2017; Topol, 2019).

The main aim of the current study was to evaluate the efficacy of the cognitive face-to-face virtual reality rehabilitation system (VRRS) and to compare it to that of face-to-face cognitive treatment as usual for subjects with MCI. We hypothesized that the face-to-face VRRS system would ameliorate memory and attentional abilities more than treatment as usual in subjects with MCI.

Moreover, we tested the hypothesis that the implementation of home-based treatment through the cognitive VRRS system could induce long-term benefits, prolonging the beneficial effects of face-to-face.

## Materials and Methods

**FIGURE 1:**
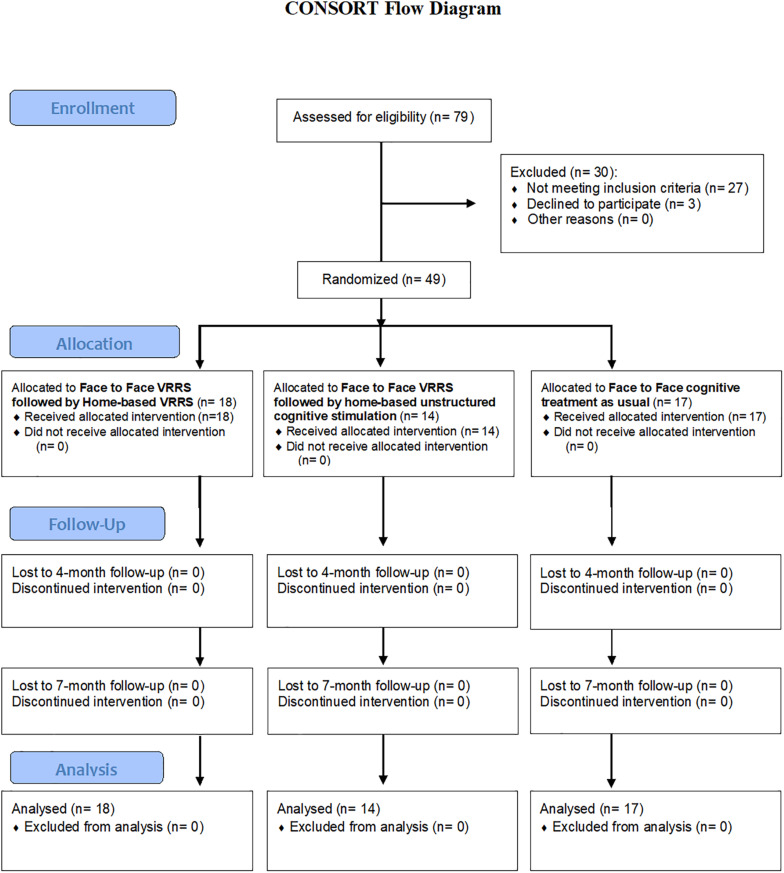
Flow chart showing study subject enrollment and sample processing.

Recruitment and treatment were conducted at the IRCCS Istituto Centro San Giovanni di Dio Fatebenefratelli of Brescia, the IRCCS Centro Neurolesi Bonino-Pulejo, Messina, and the IRCCS Fondazione Don Carlo Gnocchi Onlus of Milan, from April 2018 to February 2020 (see Figure 1).

### Study Design

This was a multicenter rater-blinded, active-controlled and randomized study. The investigators and outcome assessors were blinded to the type of treatment. Participants were randomized into three groups: (a) those who received face-to-face cognitive VRRS treatment followed by cognitive telerehabilitation (clinic-VRRS + Tele@H-VRRS), where subjects received face-to-face cognitive VRRS treatment (clinic-VRRS) followed by home-based VRRS treatment (Tele@H-VRRS); (b) those who received face-to-face cognitive VRRS treatment followed by at-home unstructured cognitive stimulation (clinic-VRRS + Tele@H-UCS), where subjects received clinic-VRRS treatment followed by at-home unstructured cognitive stimulation (Tele@H-UCS); and (c) those who received face-to-face cognitive treatment as usual (clinic-TAU), where participants received only face-to-face cognitive conventional rehabilitation.

The treatment group assigned to each patient was obtained by stratified randomization according to age and his/her performance in the Mini Mental State Examination (MMSE) (Folstein et al., 1975). Stratified randomization is achieved by generating a separate block for each combination of covariates, and participants are assigned to the appropriate block of covariates by a researcher blinded to the study aims. Details of the allocated group were given on cards contained in sequentially numbered, opaque and sealed envelopes.

The study protocol was executed in its entirety with no significant changes.

This study was approved by the local ethics committees and was conducted in accordance with the Declaration of Helsinki and reported according to CONSORT guidelines (Boutron et al., 2008; Boutron et al., 2017). The trial was registered at clinicaltrials.gov (NCT number: NCT03486704).

All participants were made fully aware of the aims of the research, and written informed consent was obtained from all subjects from the local center.

### Participants

Forty-nine older adults fulfilling the Petersen criteria for MCI (Petersen, 2011) were recruited. All participants were living independently in the community at the time of their baseline evaluation and were followed up annually during at least 2 years before recruitment in the present study.

Since no previous studies have investigated cognitive VRRS treatment effects on memory outcome, the sample size calculation was based on a prior study on patients with AD (Jelcic et al., 2014), with an effect size of 0.85 (Cohen’s d) for the MMSE score (Folstein et al., 1975) improvement after telerehabilitation treatment, a significance level (α) of 0.05 and a power (1-β) = 80 [two-tailed independent *t*-test)]. The estimated sample size was twelve participants for each group.

All the participants had normal or corrected-to-normal vision and were characterized by the following: (a) memory complaints; (b) preservation of general cognitive functioning documented by MMSE scores from 24 to 30 (Folstein et al., 1975); (c) age over 65 years; (d) global Clinical Dementia Rating (CDR) score of 0.5 (Morris, 1997); (e) preservation of functional activities; (f) absence of criteria for a diagnosis of dementia according to DSM-V (American Psychiatric Association, 2014); and (g) absence of mood and anxiety disorders. Moreover, before inclusion in the cognitive training protocol, the availability and motivation of subjects to participate consecutively in the protocol for its entire duration and the presence of a caregiver for the completion of questionnaires at all time assessments were verified.

The following stringent exclusion criteria were applied: (a) other prior or current neurological or major psychiatric disorders; (b) visual perception disorder and/or hearing loss; (c) history of traumatic brain injury, brain tumor or stroke; and (d) history of alcohol abuse. None of the participants had participated in cognitive training protocols within the year before enrollment or during the entire duration of the present study (from baseline to the last follow-up assessment).

### Assessment Procedures

#### Evaluation Timeline

The evaluations were carried out for all groups at baseline (T0), at the end of face-to-face treatment (T1, 1 month from baseline), and after 4 months (T2) and 7 months (T3) from baseline. The T2 follow-up visit corresponded to the end of the at-home treatment for the clinic-VRRS + Tele@H-VRRS and clinic-VRRS + Tele@H-UCS groups. All the assessments were carried out by expert neuropsychologists blinded to the treatment allocation of the participants. Since the participants were aware of the type of intervention received, they were advised not to declare the type of treatment carried out during the post-treatment and follow-up evaluations, making the treatment conditions unknown to the clinical psychologist who conducted the evaluations.

A comprehensive clinical, functional and neuropsychological evaluation was performed at all visits (T0, T1, T2, and T3). During baseline assessment, family history of dementia, record of medical events, current medication and complete neurologic examination results were recorded and the CDR scale (Morris, 1997) and the Cognitive Reserve Index questionnaire (CRIq) (Nucci et al., 2012) were completed. In addition, the participants in the clinic-VRRS + Tele@H-VRRS and clinic-VRRS + Tele@H-UCS groups underwent a computerized cognitive assessment and an assessment of system usability.

#### Clinical and Functional Assessment

The evaluation of subjective memory complaints was conducted using the 20-item version (range: 20–180) of the Everyday Memory Questionnaire (Sunderland et al., 1986; Calabria et al., 2011). Functional abilities were evaluated using basic (BADL) and instrumental activity of daily living (IADL) scales (Katz, 1983; Lawton and Brody, 1988). Depression was assessed with the 30-item version of the Geriatric Depression Scale (GDS) (Yesavage et al., 1982), whereas neuropsychiatric symptoms were recorded using the Neuropsychiatric Inventory (NPI) (Cummings et al., 1994; Binetti et al., 1998). Finally, quality of life was recorded using the Quality of Life in Alzheimer’s Disease (QOL-AD) scale (Bianchetti et al., 2017).

#### Neuropsychological Assessment

In addition to clinical and functional assessments, all participants were tested at each visit with a standardized neuropsychological battery. Cognitive tests were applied to assess a broad range of cognitive abilities commonly affected by MCI. The battery took approximately 90 min and included the MMSE (Folstein et al., 1975) for the assessment of global cognition; Raven’s Colored Progressive Matrices for non-verbal reasoning (Basso et al., 1987); verbal fluency (phonemic, FPL; and semantic, FPC) (Novelli et al., 1986) and action and object naming subtests from the battery for the assessment of aphasic disorders (BADA, Miceli et al., 1994) for language production; the Rey–Osterrieth complex figure test-copy (Caffarra et al., 2002) and the Clock Drawing Test (CDT) (Shulman et al., 1993) for visuo-constructional abilities; the Trail Making Test (TMT) part A and part B (Giovagnoli et al., 1996; Siciliano et al., 2019) for attention functions; and the Rey Auditory Verbal Learning Test (RAVLT), immediate and delayed recall (Carlesimo et al., 1996), the Free and Cued Selective Reminding Test (FCSRT) (Frasson et al., 2011) and the Rey–Osterrieth complex figure test-recall (Caffarra et al., 2002) for episodic memory. All the tests were administered and scored according to standard procedures (Lezak et al., 2012) (see Tables 3, 4 for details).

#### Computerized Cognitive Tasks

In the case of the participants who received face-to-face VRRS treatment (the clinic-VRRS + Tele@H-VRRS and clinic-VRRS + Tele@H-UCS groups), the performances achieved during VRRS treatment were further analyzed to assess memory, visuospatial abilities, attention and executive functions. In particular, we recorded the performance of each patient in the first clinic-VRRS session (as the baseline score, T0), and then we registered the participants’ performances obtained during the last clinic-VRRS session as the post-treatment rating (T1). Moreover, participants underwent computerized cognitive assessment during follow-up assessments (T2, T3) to analyze long-term effects.

#### Assessment of System Usability

To record subjective assessments of the clinic-VRRS, we administered the System Usability Scale (SUS) (Brooke, 1996; Bangor et al., 2008, 2009; Peres et al., 2013) to all the subjects who received clinic-VRRS + Tele@H-VRRS and clinic-VRRS + Tele@H-UCS at T1. Moreover, we recorded the SUS scores at T2 in the clinic-VRRS + Tele@H-VRRS group to assess cognitive telerehabilitation usability (Tele@H-VRRS usability). The SUS is a 10-item, five-point Likert scale (1 = strongly disagree, 5 = strongly agree). The scoring instructions described by Brooke (35) were considered. The final score ranges from 10 to 100.

#### Cognitive Rehabilitation Procedures

The cognitive rehabilitation protocol was delivered to all participants according to the corresponding experimental group.

#### Face-to-Face Cognitive VRRS Treatment (Clinic-VRRS)

Participants assigned to the clinic-VRRS group received twelve 60-min sessions of individualized cognitive rehabilitation using VRRS^1^ in the clinic with a user-centered approach over 4 weeks. Subjects were seated in front of a computer screen in a quiet room.

Face-to-face cognitive VRRS treatment included twelve exercises designed to enhance memory, visuospatial abilities, attention and executive functions (listed in Table 1). In each treatment session, the participant worked with six exercises, 10 min each, so that each exercise was completed six times over the twelve clinic-VRRS sessions. The subject was asked to continue to perform each task until the end of the set time. The therapist suggested to the participant some strategies aiming to improve performance in all the treatment sessions except for the first and last sessions. At the end of each training session, subjects received feedback on their performances, and a detailed report of the results was made available to the therapist, allowing the monitoring of progress over time. Clinical VRRS treatment was tailored to the patient’s baseline characteristics through a pre-training session. The starting level and the number of trials were adjusted according to the subject’s performance level using an adaptive staircase procedure. Progress was continuously monitored, and the exercises adaptively progressed in difficulty.

**TABLE 1:** Face to face cognitive VRRS treatment (clinic-VRRS).

Main domain	Task and description	Task duration
Memory	- *Safe opening – forward:* A sequence of numbers appears on the screen and the subject is requested to memorize it. When it disappears, the numbers must be typed on the screen in the same order of presentation to open a safe;	10 min
	- *Visual memory:* Pairs of geometric shapes or animals’ cards are displayed on the screen and the subject is requested to memorize them. When cards turn over, the subject is requested to remember the position of each pair of cards;	10 min
	- *Safe opening- backward:* A sequence of numbers appears on the screen and the subject is requested to memorize it. When it disappears, the numbers must be typed on the screen in the reverse order to open a safe;	10 min
	- *Verbal memory:* A list of words appears on the screen and the subject is requested to memorize it. When words disappear, the subject is requested to identify them in a list of many other words.	10 min
Attention and Executive functions	- *Complete the sequence of shapes:* A sequence of shapes is displayed on the screen and the subject is requested to continue the sequence, selecting the correct elements;	10 min
	- *Change color:* A geometric figure appears on the screen and the subject is requested to select, from a series of figures, the one that differs from the target only for color;	10 min
	- *Rotation:* An animal or an arrow picture is shown and the subject is requested to complete the sequence, selecting the correct pictures from a series of elements, according to the displayed direction of rotation;	10 min
	- *Complete the logical relationship:* A cuisenaire rod and a comparison operator are displayed on the screen and the subject is asked to select, from a set of colored rods, the cuisenaire rod that satisfy the logical relationship.	10 min
Visuospatial abilities	- *Spatial orientation:* A duck appears on the screen and the subject is requested to place four colored balls around the duck, following the written spatial indications and taking into account the duck’s orientation;	10 min
	- *Road route:* A road map is displayed on the screen and a ball runs a route. The subject has to pay attention to the ball and to reproduce the route;	10 min
	- *Find the symmetrical:* An animal’s picture and a rotation axis are shown and the subject is asked to select, from a series of figures, the symmetrical one;	10 min
	- *Recognize farm animals:* A farm picture is displayed and the subject is requested to find a series of farm animals.	10 min

#### Face-to-Face Cognitive Treatment as Usual (Clinic-TAU)

Participants assigned to the clinic-TAU group received twelve 60-min sessions of group cognitive stimulation in the clinic. During these group sessions, which were led by mental health professionals, reality orientation therapy, reminiscence therapy, paper and pencil exercises and metacognitive training aiming to learn cognitive strategies and to use external aids were proposed.

At the end of this face-to-face treatment, participants were requested not to perform any cognitive activity until the conclusion of the entire protocol.

#### Home-Based Cognitive VRRS Treatment (Tele@H-VRRS)

The participants assigned to the Tele@H-VRRS group received, after the end of face-to-face treatment, thirty-six 60-min sessions of home-based cognitive VRRS treatment (see “text footnote 1”), three sessions for week over 3 months. Twelve exercises designed to enhance memory, visuospatial abilities, attention and executive functions, different from those used in face-to-face VRRS training, were selected (listed in Table 2). In each treatment session, a participant worked with six exercises, 10 min each, task difficulty adaptively progressed, and the performances were continuously monitored by the therapist. The subject was asked to continue to perform each task until the end of the set time.

**TABLE 2:** At home-based VRRS treatment exercises (Tele@H-VRRS).

Main domain	Task and description	Task duration
Memory	- *Recognize banknotes and coins*: A series of banknotes and coins is presented on the screen and the subject is requested to select the one that corresponds to the requested written quantity;	10 min
	- *Collect money up to 10 euros*: A group of banknotes and coins is displayed on the screen and the subject is invited to collect coins and banknotes needed to reach the required amount of money. The maximum amount of money is 10 euros;	10 min
	- *Recognize banknotes and coins – back:* The back of a series of banknotes and coins is presented on the screen and the subject is requested to select the one that corresponds to the requested written quantity;	10 min
	- *Collect money up to 100 euros:* Banknotes and coins is displayed on the screen and the subject is invited to collect coins and banknotes needed to reach the required amount of money. The maximum amount of money is 100 euros.	10 min
Attention and Executive functions	- Change of shape: A geometric figure appears on the screen and the subject is requested to select, from a series of figures, the one that differs from the target only for shape;	10 min
	- Find the missing cuisenaire rod: A cuisenaire rod is displayed on the screen and the subject is asked to select, from a set of colored rods, the one that logically completes the sequence;	10 min
	- Change all: A geometric figure appears on the screen and the subject is requested to select, from a series of figures, the one that differs from the target for color, shape and dimension as compared to the target;	10 min
	- Complete the sequence following the rule: A cuisenaire rod and a rule are displayed on the screen. The subject is asked to select, from a set of colored rods, the two that complete the sequence, taking into account the rule displayed (ascending or descending order).	10 min
Visuospatial abilities	- *Spatial orientation-Front or rear:* A duck on a spatial axis and an animal’s picture appear on the screen. The subject is requested to indicate whether the animal’s picture appears in front or behind the duck, considering the duck’s orientation;	10 min
	- *Indicates the rotation:* A sequence of rotated animals or arrow pictures is shown and the subject has to indicate whether the direction of rotation of the elements in the sequence is clockwise or counterclockwise;	10 min
	- *Puzzle:* Individual pieces of the puzzle are shown on the screen and the subject is requested to arrange them in order to compose the whole puzzle;	10 min
	- *Connections of points:* A series of circles with numbers or letters circles are randomly presented on the screen and the subject is invited to connect the numbered or letters circles in the correct sequence, following the numerical or alphabetical order.	10 min

The VRRS system has telerehabilitation functionalities, allowing the use of the same functionalities applied in the face-to-face treatment. For the at-home treatment, each participant received a home-based kit including a tablet that allowed access to a daily individualized training program, a detailed VRRS tablet manual, an exercise instructions booklet and a participant diary. Before beginning the home-based treatment, the therapist downloaded all 36 sessions of the appropriate individualized cognitive training exercise on the patient’s tablet and assisted the participants and their caregivers at first access to familiarize them with the technological device. During home-based treatment, the therapist provided continuous assistance for technical difficulties and individualized cognitive training exercises were adjusted by the therapist once a week.

#### Home-Based Unstructured Cognitive Stimulation (Tele@H-UCS)

Subjects assigned to the Tele@H-UCS group were requested to work on detailed activities 60 min a day, three times a week (36 sessions in total) over 12 weeks after the end of face-to-face treatment. They received, from the therapist, an instructions booklet and a participant diary. Conventional instruments, such as paper and pencil exercises, creative manual activities, reading newspapers and magazines, watching documentaries, crosswords and sudoku, were suggested. Each participant was requested to compile a detailed diary reporting the performed activities.

### Outcomes

Primary outcome measures were the changes in two tasks of verbal episodic memory: (a) the RAVLT, immediate and delayed recall; and (b) the FCSRT.

The secondary outcomes included quality of life measures, subjective memory complaints and neuropsychological measures evaluating memory, language, attention and visuo-constructional functions. The choice of outcomes was based on previous literature findings and on predefined hypotheses of investigation. Inference on them was performed considering each outcome singularly (i.e., not simultaneous inference was done on all outcome, thus no multiple comparison was needed to compare outcomes results each other (see e.g., Proschan and Follmann, 1995; Bender and Lange, 2001).

### Statistical Analysis

Summary statistics are expressed as means and standard deviations. Comparisons of socio-demographic features between groups were evaluated by parametric (*t*-tests) or corresponding non-parametric (Mann–Whitney) tests. Variable distribution was inspected through histograms and the use of the Kolmogorov–Smirnov and Shapiro–Wilk tests. Consistent with the type of variable distribution (Gaussian, Poisson or Gamma), generalized linear mixed models (GLMMs) were performed to evaluate score differences across time points and between groups. In detail, the first series of GLMMs was applied to compare two groups (clinic-TAU vs. clinic-VRRS + Tele@H-VRRS together with clinic-VRRS + Tele@H-UCS) across two time points (baseline and post-treatment). Subsequently, GLMMs were performed to compare the three subject groups across four time points (baseline, post-treatment, 4-month follow-up, and 7-month follow-up). Different test scores were used as dependent variables (one for each model), and the effects (time, group and time × group) were considered independent variables. Sequential Sidak correction was used for the evaluation of *post hoc*.

Statistical significance was set at *p* < 0.05. Statistical analyses were carried out by using SPSS 25.0. Graphs were generated using R software (R Core Team, 2013).

## Results

A total of 79 subjects were evaluated for inclusion in this study. Ultimately, 30 subjects were excluded (27 subjects did not meet the inclusion criteria, and 3 subjects declined to participate), whereas 49 subjects were deemed eligible for participation.

These 49 subjects were randomized into the three experimental groups: 18 participants were allocated to the clinic-VRRS + Tele@H-VRRS group, 14 subjects to the clinic-VRRS + Tele@H-UCS group and 17 participants to the clinic-TAU group. All the participants completed the baseline (T0), post-treatment (T1) and follow-up (T2 and T3) evaluations (see Figure 1-Flowchart).

Adherence to treatment was indexed by the number of sessions completed for each participant. Concerning the clinic-VRRS + Tele@H-VRRS group, all 18 participants completed the 12 sessions of face-to-face VRRS treatment, 6 participants completed all 36 sessions of at-home VRRS training, whereas all other subjects completed more than 70% of the telerehabilitation sessions. Moreover, all 14 participants in the clinic-VRRS + Tele@H-UCS group completed the 12 sessions of face-to-face VRRS treatment, 7 subjects completed the 36 sessions of at-home unstructured cognitive stimulation, and the other subjects completed more than 70% of the at-home unstructured cognitive stimulation sessions. Finally, 8 subjects in the clinic-TAU group completed the 12 sessions, whereas all the other subjects completed more than 70% of the usual treatment sessions.

### Participants

We enrolled 49 patients with MCI, 42 (86%) amnestic MCI and 7 (14%) non-amnestic MCI participants. In particular, the current sample included: (i) 20 amnestic single domain MCI (aMCI-s); (ii) 22 amnestic multiple domain MCI (aMCI-m); 10 of them showed an additional deficit in visuo-constructional functions, 2 in attention, 3 in language, 4 in attention and visuo-constructional functions and 1 in attention and language; (iii) 6 non-amnestic single domain MCI (naMCI-s), presence of a disability in another cognitive area (1 of them showed deficit in language, 3 in visuo-constructional functions and 2 in attention abilities), with normal memory; and (iv) 1 non-amnestic multiple domain MCI (naMCI-m), with disabilities in attention and visuo-constructional functions, with normal memory.

The mean age of the participants was 76.5 years (SD = 4.2), the mean number of years of education was 10.7 years (SD = 4.4), and 24 participants (49.0%) were male. The clinic-VRRS + Tele@H-VRRS group included 13 male and 5 female participants (age: mean, 75.3 years, SD: 3.3; education: 11.8 years, SD 4.8); the clinic-VRRS + Tele@H-UCS group included 4 male and 10 female participants (age: mean, 76.3 years, SD: 4.9; education: 10.5 years, SD 4.8); and the clinic-TAU group included 7 male and 10 female participants (age: mean, 78.1 years, SD: 4.1; education: 9.8 years, SD 3.7).

The three groups did not differ regarding age (*p* = 0.142) or education (*p* = 0.405), but there was a significant difference in sex (*p* = 0.036). Descriptive statistics of all clinical features of the three groups of patients, measured at the four time points, are reported in Table 3.

**TABLE 3:** Descriptive statistics results for Neuropsychological assessment.

	Face to Face VRRS followed by home-based VRRS (*n* = 18)				Face to Face VRRS followed by Home-based unstructured cognitive stimulation (*n* = 14)				Face to Face cognitive treatment as usual (*n* = 17)				
	Baseline	Post-treatment	4 Months Follow-up	7 Months Follow-up	Baseline	Post-treatment	4 Months Follow-up	7 Months Follow-up	Baseline	Post-treatment	4 Months Follow-up	7 Months Follow-up	Cut-off

	Mean (SD)	Mean (SD)	Mean (SD)	Mean (SD)	Mean (SD)	Mean (SD)	Mean (SD)	Mean (SD)	Mean (SD)	Mean (SD)	Mean (SD)	Mean (SD)	
**Clinical and Functional Assessment**													
Everyday memory Questionnaire (EMQ)	67.8 (22.9)	62.3 (24.4)	64.5 (27.9)	61.0 (24.1)	76.6 (30.9)	76.0 (30.6)	76.2 (30.0)	72.4 (26.8)	66.2 (27.1)	61.9 (26.3)	62.0 (26.5)	71.1 (25.2)	
**Quality of Life in Alzheimer’s Disease (QOL-AD)**													
QOL-AD – Composite score	34.4 (4.0)	34.3 (4.2)	34.9 (4.0)	32.6 (8.2)	33.0 (5.9)	33.4 (5.7)	32.1 (6.9)	32.3 (7.3)	34.9 (4.4)	35.1 (3.4)	34.7 (4.2)	32.7 (4.9)	>33
**Memory**													
Rey Auditory Verbal Learning Test (RAVLT), immediate recall	29.2 (6.8)	29.4 (7.4)	29.9 (8.6)	26.1 (7.6)	31.0 (7.0)	32.1 (6.7)	30.2 (8.1)	33.4 (8.4)	30.2 (7.7)	30.1 (6.5)	30.9 (6.6)	31.5 (7.8)	>28.52
Rey Auditory Verbal Learning Test (RAVLT), delayed recall	4.0 (3.1)	4.3 (3.3)	3.9 (3.9)	3.6 (3.3)	4.6 (2.3)	4.3 (3.4)	5.1 (3.6)	6.0 (4.2)	4.5 (3.2)	4.2 (3.0)	4.2 (2.6)	4.8 (3.9)	>4.68
**Free and Cued Selective Reminding Test (FCSRT)**													
FCSRT – Immediate free recall (IFR)	18.4 (7.7)	20.6 (9.4)	18.2 (8.5)	17.8 (8.0)	20.4 (6.1)	24.4 (6.5)	22.1 (6.3)	21.4 (6.2)	19.7 (7.9)	19.9 (9.0)	21.4 (8.4)	21.7 (10.2)	>19.59
FCSRT – Immediate Total Recall (ITR)	32.0 (4.3)	32.1 (5.5)	31.2 (6.5)	32.0 (4.4)	33.4 (4.9)	33.6 (3.8)	33.9 (3.2)	34.2 (3.1)	33.4 (3.2)	32.5 (4.9)	33.1 (3.9)	33.5 (3.6)	≥35
FCSRT – Delayed Free Recall (DFR)	5.8 (3.2)	6.6 (3.5)	5.7 (3.6)	6.6 (4.1)	6.7 (2.8)	8.3 (2.6)	8.6 (3.2)	8.1 (3.2)	6.4 (4.3)	7.0 (3.8)	6.6 (4.0)	7.2 (4.1)	>6.31
FCSRT – Delayed total recall (DTR)	10.4 (2.1)	10.3 (2.5)	10.4 (1.8)	10.4 (2.0)	10.9 (1.9)	11.1 (1.6)	11.3 (1.5)	11.4 (1.2)	10.8 (2.1)	10.9 (1.9)	10.8 (1.5)	11.2 (1.2)	≥11
FCSRT – Index of sensitivity of cueing (ISC)	0.8 (0.2)	0.8 (0.2)	0.8 (0.2)	0.8 (0.2)	0.9 (0.2)	0.8 (0.2)	0.9 (0.1)	0.9 (0.1)	0.9 (0.1)	0.8 (0.2)	0.9 (0.2)	0.9 (0.1)	≥0.9
**Language**													
Verbal fluency, phonemic (FPL)	29.7 (7.1)	31.5 (9.1)	29.2 (6.6)	30.1 (7.6)	29.8 (6.7)	33.4 (8.4)	30.6 (8.7)	29.8 (9.5)	28.9 (8.4)	31.7 (8.8)	31.2 (11.5)	30.4 (7.9)	>16
Verbal fluency, semantic (FPC)	27.8 (5.8)	30.8 (6.8)	30.1 (5.5)	29.1 (6.4)	29.6 (5.9)	29.2 (6.8)	28.5 (3.9)	27.8 (4.6)	30.9 (6.3)	29.4 (6.0)	29.1 (7.8)	29.2 (6.1)	>24
**Battery for Analysis of Aphasic Deficits (B.A.D.A.)**													
B.A.D.A. – Objects naming	26.8 (2.4)	27.1 (2.2)	26.7 (2.4)	26.9 (2.5)	26.4 (1.7)	27.0 (2.5)	26.4 (2.6)	27.2 (1.7)	26.9 (2.3)	27.1 (1.8)	27.2 (1.8)	27.8 (2.2)	
B.A.D.A. – Actions naming	24.5 (3.0)	25.2 (2.4)	24.9 (2.5)	24.9 (2.8)	24.1 (2.4)	24.6 (3.0)	24.4 (3.0)	25.0 (2.8)	24.4 (2.9)	25.6 (2.4)	25.1 (2.3)	25.5 (2.6)	
**Attentional functions**													
**Trail Making Test (TMT)**													
TMT, part A (msec)	58.4 (29.0)	52.0 (25.3)	58.1 (24.7)	55.3 (24.1)	59.5 (21.2)	53.9 (17.7)	65.9 (26.1)	64.5 (27.2)	46.8 (16.0)	47.1 (16.2)	52.4 (21.4)	60.0 (37.9)	<94
TMT, part B (msec)	251.3 (146.3)	216.6 (140.0)	219.1 (139.4)	245.3 (126.4)	237.6 (160.0)	238.3 (119.7)	241.4 (142.2)	276.7 (118.5)	206.9 (123.8)	231.2 (184.7)	219.8 (164.3)	268.2 (201.5)	<283
**Visuo-constructional functions**													
Clock Drawing Test (CDT)	2.0 (0.8)	1.7 (0.8)	1.7 (0.9)	1.7 (0.8)	2.9 (1.0)	2.2 (1.1)	2.6 (1.2)	2.6 (0.9)	1.9 (0.7)	1.8 (0.7)	2.0 (1.1)	1.8 (0.7)	≤2

*Raw scores mean are reported. Standard deviation between brackets. Cut-off scores according to Italian normative data are reported. msec: milliseconds; VRRS: virtual reality rehabilitation system.*

### Face-to-Face Cognitive Virtual Reality Rehabilitation System vs. Face-to-Face Cognitive Treatment as Usual

Our first aim was to evaluate the efficacy of the face-to-face VRRS (clinic-VRRS) and to compare it to that of cognitive treatment as usual (clinic-TAU). For this purpose, we considered two groups of subjects: those who received VRRS (i.e., the clinic-VRRS + Tele@H-VRRS and clinic-VRRS + Tele@H-UCS groups) and those who received treatment as usual (i.e., the clinic-TAU group), and we compared their scores at the first two time points (baseline, T0; and after face-to-face treatment, T1).

We performed different GLMMs, one for each outcome, with time (two time points), group (two treatments) and their interaction as independent variables, to verify whether the two treatments had different effects on the outcomes. The results are shown in Table 4 (first columns).

**TABLE 4:** Generalized Linear Mixed Models results for neuropsychological test.

	clinic-VRRS (clinic-VRRS + Tele@H-VRRS and clinic-VRRS + Tele@H-UCS) vs. clinic-TAU longitudinal evaluation at two time points			clinic-VRRS + Tele@H-VRRS vs. clinic-VRRS + Tele@H-UCS vs. clinic-TAU longitudinal evaluation at four time points		
	p_Time	p_Time	p_Time × Group	p_Time	p_Group	p_Time × Group
**Clinical and Functional Assessment**						
Everyday Memory Questionnaire (EMQ)	**0.005**	0.450	0.713	0.072	0.412	0.288
**Quality of Life in Alzheimer’s Disease (QOL-AD)**						
QOL-AD – composite score	0.701	0.333	0.999	0.184	0.687	0.472
**Memory**						
Rey Auditory Verbal Learning Test (RAVLT), immediate recall	0.796	0.962	0.699	0.767	0.269	0.088
Rey Auditory Verbal Learning Test (RAVLT), delayed recall	0.779	0.933	0.629	0.601	0.636	0.086
**Free and Cued Selective Reminding Test (FCSRT)**						
FCSRT – Immediate free recall (IFR)	**0.004**	0.675	**0.010**	**<0.001**	0.275	**0.003**
FCSRT – Immediate total recall (ITR)	0.222	0.773	0.253	0.293	0.431	0.335
FCSRT – delayed free recall (DFR)	**0.012**	0.932	0.471	**0.003**	0.256	0.085
FCSRT – delayed total recall (DTR)	0.545	0.661	0.942	0.103	0.486	0.702
FCSRT – index of sensitivity of cueing (ISC)	0.137	0.347	0.222	**0.031**	0.219	0.829
**Language**						
Verbal fluency, phonemic (FPL)	<0.001	0.767	0.904	0.006	0.955	0.783
Verbal fluency, semantic (FPC)	0.967	0.640	**0.024**	0.536	0.880	0.085
**Battery for Analysis of Aphasic Deficits (B.A.D.A.)**						
B.A.D.A. – Objects naming	0.239	0.828	0.671	0.129	0.747	0.677
B.A.D.A. – Actions naming	**0.005**	0.630	0.289	**0.024**	0.778	0.797
**Attentional functions**						
Trail Making Test (TMT)						
TMT, part A (msec)	0.098	0.104	**0.058**	**0.002**	0.411	0.150
TMT, part B (msec)	0.836	0.686	0.176	0.315	0.900	0.549
**Visuo-constructional functions**						
Clock Drawing Test (CDT)	**0.001**	0.211	**0.010**	**0.006**	**0.005**	0.276

*msec: milliseconds; VRRS: virtual reality rehabilitation system; bold font indicates statistical significance.*

The performances of the two groups at the two time points were significantly different for the FCSRT IFR, FPC and CDT (*p*-value of the interaction term equal to 0.010, 0.024, and 0.010, respectively). Moreover, for the TMT A, there was a trend toward significance (*p* = 0.058).

**FIGURE 2:**
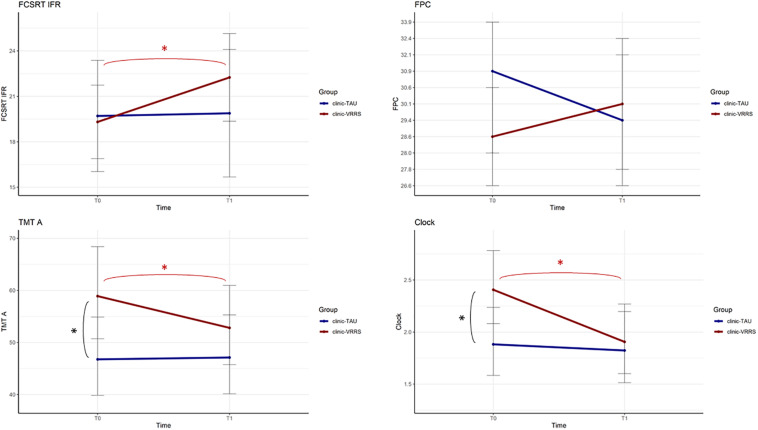
Effects of Face to Face cognitive VRRS (clinic-VRRS) vs. Face to Face cognitive treatment as Usual (clinic-TAU) on neuropsychological assessment. Asterisks indicate statistical significance.

All the resulting significant outcomes are presented in Figure 2. In particular, the FCSRT IFR, CDT and TMT A scores improved only after clinic-VRRS (FCSRT IFR: *p* < 0.001; CDT: *p* < 0.001; TMT A: *p* = 0.002). Although in TMT A and CDT scores a significant difference was observed between the two groups at baseline assessment, an improvement in these tests could be recorded exclusively in clinic-VRRS group. Regarding the FPC, the clinic-VRRS group showed an increase from T0 to T1, whereas the clinic-TAU group showed worse FPC performance, inducing a significant time × group interaction (although the changes within groups did not reach significance). Thus, by combining the information provided by the models and figures, we showed that the clinic-VRRS was more efficient than clinic-TAU, improving memory (FCSRT IFR), language (FPC), attention (TMT A) and visuo-constructional abilities (CDT).

### Home-Based VRRS Rehabilitation (Tele@H-VRRS) vs. Home-Based Unstructured Cognitive Stimulation (Tele@H-UCS) vs. No Additional Treatment

After the first month, the participants who received clinic-VRRS were divided into two subgroups in which cognitive telerehabilitation and unstructured cognitive stimulation were provided, while those who received usual care did not receive any additional treatment during the following months. Our second aim was to assess whether the telerehabilitation VRRS system could extend the beneficial effects of face-to-face VRRS treatment.

To verify this hypothesis, different GLMMs were performed (one for each outcome) using time (four time points), group (three groups) and their interaction as independent variables. The results of the models are shown in Table 4 (last columns).

Regarding the neuropsychological assessment, the outcome that behaved in a significantly different way in the three groups over time was the FCSRT IFR. As reported earlier, the FCSRT IFR showed an improvement from T0 to T1 in the clinic-VRRS group. By splitting the clinic-VRRS group into two subgroups, this improvement significantly decreased after the end of face-to-face treatment in the clinic-VRRS + Tele@H-UCS group (T1 vs. T2 *p* = 0.025; T1 vs. T3 *p* = 0.003), whereas no strong evidence of changes in the clinic-VRRS + Tele@H-VRRS group was recorded (T1 vs. T2 *p* = 0.055; T1 vs. T3 *p* = 0.084). Although the mean changes from T1 to T2 were similar in clinic-VRRS + Tele@H-UCS and in clinic-VRRS + Tele@H-VRRS groups, different statistical significance were mainly driven by difference in variability (standard deviations) recorded in the two experimental groups. As stated above, the clinic-TAU group did not show any significant modification across all time points (see Table 4).

### Computerized Cognitive Tasks

**FIGURE 3:**
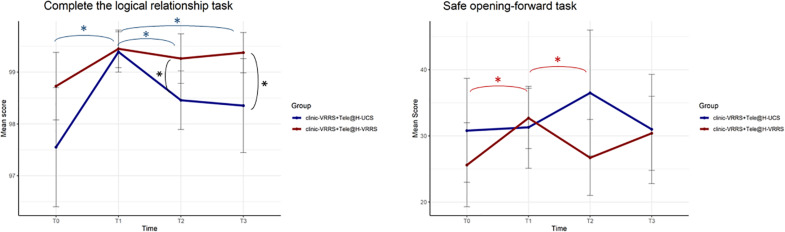
Effects of home-based cognitive telerehabilitation (Tele@H-VRRS) vs. home-based unstructured cognitive stimulation (Tele@H-UCS) vs. no treatment on computerized cognitive tasks. Asterisks indicate statistical significance.

Different GLMMs were performed (Table 5), and it followed that the two groups were different, across time, in the two tasks “Complete the logic relationship” and “Safe opening-forward” (interaction term *p*-values equal to 0.007 and 0.016, respectively). For the first task, the performance of subjects was different at all time points in the two groups (Figure 3). *Post hoc* analysis showed that there was a different trend in the clinic-VRRS + Tele@H-VRRS and clinic-VRRS + Tele@H-UCS groups, with very similar scores at T1 (*p* = 0.825) and higher scores in the clinic-VRRS + Tele@H-VRRS group than in the clinic-VRRS + Tele@H-UCS group at both follow-up visits (T2: *p* = 0.033; T3: *p* = 0.042). For the safe opening-forward task, the score differed significantly over time between the two groups: only the clinic-VRRS + Tele@H-VRRS group showed an improvement from T0 and T1 (*p* = 0.017) and worsening from T1 and T2 (*p* = 0.044).

**TABLE 5:** Descriptive statistics and Generalized Linear Mixed Models results for Computerized Cognitive Tasks.

	Face to Face VRRS followed by home-based VRRS (*n* = 18)*				Face to Face VRRS followed by home-based unstructured cognitive stimulation (*n* = 14)						
			
	Baseline	Post-treatment	4 Months Follow-up	7 Months Follow-up	Baseline	Post-treatment	4 Months Follow-up	7 Months Follow-up	p_Time	p_Group	p_Time × Group

	Mean (SD)	Mean (SD)	Mean (SD)	Mean (SD)	Mean (SD)	Mean (SD)	Mean (SD)	Mean (SD)			
**Memory**											
**Safe opening – forward**											
Mean score (%)	25.6 (13.2)	32.7 (9.4)	26.7 (11.6)	31.7 (11.3)	30.8 (15.5)	31.3 (12.1)	36.5 (18.0)	31.0 (14.5)	**0.047**	0.418	**0.016**
**Visual memory**											
Mean score (%)	80.0 (4.7)	80.0 (5.5)	80.1 (7.1)	78.2 (6.2)	79.7 (4.8)	81.3 (3.7)	79.8 (3.3)	81.5 (3.4)	0.827	0.41	0.168
**Safe opening – backward**											
Mean score (%)	29.6 (11.2)	33.7 (9.5)	32.3 (13.8)	37.0 (21.1)	30.6 (14.9)	36.3 (9.8)	36.9 (8.1)	37.1 (12.8)	**0.021**	0.547	0.690
**Verbal memory**											
Mean score (%)	82.6 (6.5)	84.4 (6.4)	84.9 (6.0)	84.4 (5.1)	81.5 (5.6)	85.5 (3.9)	85.8 (4.9)	84.4 (4.9)	**0.010**	0.993	0.484
**Attention and Executive functions**											
**Complete the sequence of shapes**											
Mean score (%)	93.1 (7.8)	97.3 (2.8)	97.6 (2.3)	97.2 (3.3)	91.9 (9.1)	96.7 (3.7)	97.6 (2.0)	97.5 (2.7)	**0.004**	0.751	0.914
**Change color**											
Mean score (%)	99.6 (0.3)	99.9 (0.2)	99.9 (0.2)	99.9 (0.1)	99.4 (0.3)	99.8 (0.2)	99.8 (0.2)	99.7 (0.4)	**<0.001**	**0.021**	0.205
**Rotation**											
Mean score (%)	90.6 (4.4)	94.4 (4.9)	90.3 (16.9)	89.0 (19.7)	85.7 (5.9)	89.0 (8.6)	87.2 (9.5)	88.7 (8.5)	**<0.001**	0.224	0.665
**Complete the logical relationship**											
Mean score (%)	98.7 (1.4)	99.4 (0.8)	99.3 (1.0)	99.2 (0.9)	97.6 (2.3)	99.4 (0.8)	98.5 (1.0)	98.5 (1.6)	**<0.001**	**0.019**	**0.007**
**Visuospatial abilities**											
**Spatial orientation**											
Mean score (%)	98.4 (1.9)	99.2 (0.8)	98.8 (1.4)	99.2 (1.2)	97.0 (3.2)	99.1 (0.7)	98.4 (1.4)	98.4 (1.6)	**0.009**	0.101	0.403
**Road route**											
Mean score (%)	84.8 (7.6)	90.4 (4.3)	86.2 (4.3)	88.0 (6.8)	85.2 (4.4)	91.0 (4.9)	86.0 (5.8)	85.4 (6.2)	**<0.001**	0.679	0.610
**Find the symmetrical**											
Mean score (%)	95.3 (1.7)	95.7 (1.2)	96.2 (0.9)	95.6 (1.3)	94.0 (1.2)	94.6 (1.0)	94.6 (1.1)	94.7 (0.8)	**0.037**	<0.001	0.470
**Recognize farm animals**											
Mean score (%)	86.5 (10.3)	92.4 (3.6)	91.7 (4.3)	88.1 (5.7)	82.7 (7.4)	92.4 (5.2)	89.9 (6.8)	86.9 (7.6)	**<0.001**	0.288	0.370

*data available for 16 patients of this group. Standard deviation between brackets. msec: milliseconds; VRRS: virtual reality rehabilitation system; bold font indicates statistical significance.

### System Usability Scale

Interestingly, the SUS, administered at T1 to the two groups who received clinic-VRRS, showed good usability performance of the clinic-VRRS system (67.8, SD 11.6), and the SUS scores obtained at T2 in the clinic-VRRS + Tele@H-VRRS group, which received home-based telerehabilitation from T1 and T2, highlighted good usability performance from the VRRS telerehabilitation system (69.6, SD 8.8) (Bangor et al., 2008).

## Discussion

In recent years, the potential usefulness of cognitive training in normal aging and MCI has received increased attention (Brown, 2015; Vannini et al., 2017). Recent meta-analyses and reviews have reported that non-pharmacological cognitive interventions are effective in maintaining cognitive function in high-risk older adults (Kidd, 2008; Li et al., 2011, 2014, 2017; Hong et al., 2015; Janoutova et al., 2015; Sherman et al., 2017; Yao et al., 2020).

Different strategies, including stimulation restorative cognitive training, compensatory cognitive training and multicomponent training, have been used to reduce cognitive difficulties in subjects with MCI when assessing different outcomes (for review see Li et al., 2011; Hong et al., 2015; Sherman et al., 2017).

The main purpose of this study was to investigate the efficacy of the face-to-face cognitive VRRS and to compare it to that of face-to-face cognitive treatment as usual for subjects with MCI. Specifically, we hypothesized that face-to-face cognitive VRRS treatment may lead to an improvement in cognitive functions, that is, memory and attentional abilities, compared with face-to-face cognitive treatment as usual.

Moreover, we assessed whether an innovative cognitive telerehabilitation program could induce long-term cognitive benefits.

To address these questions, we compared the effects of face-to-face cognitive VRRS followed by cognitive telerehabilitation (clinic-VRRS + Tele@H-VRRS), face-to-face cognitive VRRS followed by at-home unstructured cognitive stimulation (clinic-VRRS + Tele@H-UCS), and face-to-face cognitive rehabilitation program as usual (clinic-TAU) on cognition in patients with MCI.

Overall, high rates of participant agreement, recruitment and treatment adherence supported the feasibility of both face-to-face and telerehabilitation interventions. Moreover, the analyses on system usability evidenced good usability of clinic-VRRS and Tele@H-VRRS.

The current findings show a significant improvement in memory, language and visuo-constructional abilities after the end of face-to-face cognitive VRRS treatment compared to face-to-face cognitive treatment as usual. Our findings are in line with previous studies that described the usefulness of face-to-face virtual reality rehabilitation protocols aimed at improving memory and executive functioning in older participants with mild cognitive difficulties (Liao et al., 2019; Moreno et al., 2019; Tuena et al., 2020) as well as previous studies of effective cognitive treatments to prevent and slow the progression of MCI (Belleville, 2008).

Regarding at-home treatment, we found that cognitive VRRS telerehabilitation has comparable effects to conventional rehabilitation in improving cognitive abilities in patients with neurodegenerative diseases. We evidenced positive effects of VRRS telerehabilitation interventions, generally comparable with those cognitive unstructured home-based intervention. This comparable effect may be related to the modalities in which the VRRS intervention was delivered: a computerized cognitive training modality without a real interaction and feedback between patient and therapist. The synchronous (in which patient and therapist perform exercises in real time) or asynchronous (in which patient and therapist do not interact in real time) interactions, and the type of monitoring feedback, i.e., on line (during the intervention) or off line (delayed), are key components that may influence the efficacy of the telerehabilitation and have thus been considered (Di Tella et al., 2019) in future studies. Importantly, clinic-VRRS + Tele@H-VRRS group showed greater maintenance of treatment gains in episodic memory (i.e., FCSRT). Moreover, participants in the clinic-VRRS + Tele@H-VRRS group exhibited significant intervention-related improvements in executive functions as assessed by computerized cognitive tasks, with maintenance of gains at the 7-month follow-up. Our results might be related to patient’s engagement and adherence in a telerehabilitation design involving asynchronous therapist-patient interactions (Matamala-Gomez et al., 2020).

The available evidence is insufficient to draw firm conclusions on the effects of different interventions on functional activities or quality of life. Further, well-designed studies investigating the efficacy of cognitive telerehabilitation are necessary.

We acknowledge that our study has some limitations. First, given our relatively small sample size, the recorded findings should be confirmed in larger samples to reach a firm conclusion. A larger sample size would make it possible to take into account in the analyses individual variables such as age, sex and gender. Furthermore, the adoption of longer follow-up visits in future studies would better highlight the duration of the long-term effects induced by the applied treatments. Finally, in the present study some intergroup variabilities in cognitive scores are recorded and the use of a crossover design could be used in future studies in order to avoid problems of comparability of the experimental groups with regard to confounding characteristics.

Notwithstanding this, our study provides preliminary evidence in support of individualized VRRS treatment, and telerehabilitation delivery for cognitive rehabilitation is quite encouraging and should pave the way for future studies aiming at identifying optimal treatment protocols in subjects with MCI. This research supports the feasibility and benefits of cognitive rehabilitation provided by telerehabilitation systems. This may be particularly important for subjects with limited access to therapy due to geographical distance, transport difficulties or a lack of local services. The recent COVID-19 emergency has clearly indicated the importance of remote delivery of cognitive rehabilitation to support ongoing rehabilitation services and guarantee continuity of care to subjects with cognitive impairment.

## Data Availability Statement

The raw data supporting the conclusions of this article will be made available by the authors, without undue reservation.

## Ethics Statement

The studies involving human participants were reviewed and approved by Comitato Etico IRCCS Istituto Centro San Giovanni di Dio–Fatebenefratelli; 25125 BRESCIA–Via Pilastroni, 4. The patients/participants provided their written informed consent to participate in this study.

## Author Contributions

RM, FB, FA, SI, PB, SC, and MC: conception and methodology. AM and CF: formal analysis. RM, EG, FB, IP, FR, SD, FA, VC, GB, SI, PB, SC, and MC: investigation. RM, EG, FB, AM, CF, IP, FR, SD, FA, VC, GB, and MC: data curation. RM, EG, AM, CF, IP, and MC: writing – original draft preparation. All authors contributed to writing – review and editing and have read and agreed to the published version of the manuscript.

## Conflict of Interest

The authors declare that the research was conducted in the absence of any commercial or financial relationships that could be construed as a potential conflict of interest.

## Footnotes

1 http://khymeia.com/

## References

[B1] American Psychiatric Association (2014). *DSM-5: Manuale Diagnostico e Statistico dei Disturbi Mentali.* Milan: Raffaello Cortina editore.

[B2] Antonietti A. Gandolla M. Rossini M. Molteni F. Pedrocchi A. Consortium A. (2016). “Interference between cognitive and motor recovery in elderly dementia patients through a holistic tele-rehabilitation platform,” in *International Conference on Wireless Mobile Communication and Healthcare*, Vol. 192 eds Perego P. Andreoni G. Rizzo G. (Cham: Springer), 359–366. 10.1007/978-3-319-58877-3_45

[B3] Astell A. J. (2019). Technology and dementia: the future is now. *Dement. Geriatr. Cogn. Disord.* 47 129–130. 10.1159/000497799 31234175

[B4] Bangor A. Kortum P. Miller J. (2009). Determining what individual SUS scores mean: adding an adjective rating scale. *J. Usability Stud.* 4 114–123.

[B5] Bangor A. Kortum P. T. Miller J. T. (2008). An empirical evaluation of the system usability scale. *Int. J. Hum. Comput. Interact.* 24 574–594. 10.1080/10447310802205776

[B6] Basso A. Capitani E. Laiacona M. (1987). Raven’s coloured progressive matrices: normative values on 305 adult normal controls. *Funct. Neurol.* 2 189–194. 3666548

[B7] Belleville S. (2008). Cognitive training for persons with mild cognitive impairment. *Int. Psychogeriatr.* 20 57–66. 10.1017/s104161020700631x 17958927

[B8] Bender R. Lange S. (2001). Adjusting for multiple testing—when and how? *J. Clin. Epidemiol.* 54 343–349. 10.1016/s0895-4356(00)00314-0 11297884

[B9] Bharucha A. J. Anand V. Forlizzi J. Dew M. A. Reynolds C. F. III Stevens S. (2009). Intelligent assistive technology applications to dementia care: current capabilities, limitations, and future challenges. *Am. J. Geriatr. Psychiatry* 17 88–104. 10.1097/jgp.0b013e318187dde5 18849532 PMC2768007

[B10] Bianchetti A. Cornali C. Ranieri P. Trabucchi M. (2017). Quality of life in patients with mild dementia. Validation of the Italian version of the quality of life Alzheimer’s disease (QoL-AD) scale. *J. Ital. Soc. Gerontol. Geriatr.* 65:137.

[B11] Binetti G. Mega M. S. Magni E. Padovani A. Rozzini L. Bianchetti A. (1998). Behavioral disorders in Alzheimer disease: a transcultural perspective. *Arch. Neurol.* 55 539–544. 10.1001/archneur.55.4.539 9561983

[B12] Boutron I. Altman D. G. Moher D. Schulz K. F. Ravaud P. Group C. N. (2017). CONSORT statement for randomized trials of nonpharmacologic treatments: a 2017 update and a CONSORT extension for nonpharmacologic trial abstracts. *Ann. Intern. Med.* 167 40–47. 10.7326/m17-0046 28630973

[B13] Boutron I. Moher D. Altman D. G. Schulz K. F. Ravaud P. Group C. (2008). Extending the CONSORT statement to randomized trials of nonpharmacologic treatment: explanation and elaboration. *Ann. Intern. Med.* 148 295–309. 10.7326/0003-4819-148-4-200802190-00008 18283207

[B14] Brennan D. Georgeadis A. Baron C. (2002). Telerehabilitation tools for the provision of remote speech-language treatment. *Top. Stroke Rehabil.* 8 71–78. 10.1310/u7kv-dy7u-q6qp-lvbp 14523731

[B15] Brennan D. M. Mawson S. Brownsell S. (2009). Telerehabilitation: enabling the remote delivery of healthcare, rehabilitation, and self management. *Stud. Health Technol. Inform.* 145 231–248. 19592797

[B16] Brennan D. M. Tindall L. Theodoros D. Brown J. Campbell M. Christiana D. (2011). A blueprint for telerehabilitation guidelines–October 2010. *Telemed. J. E Health* 17 662–665. 10.1089/tmj.2011.0036 21790271

[B17] Brooke J. (1996). SUS-A quick and dirty usability scale. *Usability Eval. Ind.* 189 4–7.

[B18] Brown G. C. (2015). Living too long: the current focus of medical research on increasing the quantity, rather than the quality, of life is damaging our health and harming the economy. *EMBO Rep.* 16 137–141. 10.15252/embr.201439518 25525070 PMC4328740

[B19] Burdea G. Polistico K. Krishnamoorthy A. House G. Rethage D. Hundal J. (2015). Feasibility study of the BrightBrainer^TM^ integrative cognitive rehabilitation system for elderly with dementia. *Disabil. Rehabil. Assist. Technol.* 10 421–432. 10.3109/17483107.2014.900575 24679074 PMC4942847

[B20] Burton R. L. O’Connell M. E. (2018). Telehealth rehabilitation for cognitive impairment: randomized controlled feasibility trial. *JMIR Res. Protoc.* 7:e43. 10.2196/resprot.9420 29422453 PMC5824099

[B21] Caffarra P. Vezzadini G. Dieci F. Zonato F. Venneri A. (2002). Rey-Osterrieth complex figure: normative values in an Italian population sample. *Neurol. Sci.* 22 443–447. 10.1007/s100720200003 11976975

[B22] Calabria M. Manenti R. Rosini S. Zanetti O. Miniussi C. Cotelli M. (2011). Objective and subjective memory impairment in elderly adults: a revised version of the everyday memory questionnaire. *Aging Clin. Exp. Res.* 23 67–73. 10.1007/bf03324954 21499021

[B23] Carlesimo G. A. Caltagirone C. Gainotti G. (1996). The mental deterioration battery: normative data, diagnostic reliability and qualitative analyses of cognitive impairment. The group for the standardization of the mental deterioration battery. *Eur. Neurol.* 36 378–384. 10.1159/000117297 8954307

[B24] Cherney L. R. van Vuuren S. (2012). Telerehabilitation, virtual therapists, and acquired neurologic speech and language disorders. *Semin. Speech Lang.* 33 243–257. 10.1055/s-0032-1320044 22851346 PMC3691350

[B25] Cotelli M. Manenti R. Brambilla M. Gobbi E. Ferrari C. Binetti G. (2019). Cognitive telerehabilitation in mild cognitive impairment, Alzheimer’s disease and frontotemporal dementia: a systematic review. *J. Telemed. Telecare* 25 67–79. 10.1177/1357633x17740390 29117794

[B26] Cummings J. L. Mega M. Gray K. Rosenberg-Thompson S. Carusi D. A. Gornbein J. (1994). The neuropsychiatric inventory: comprehensive assessment of psychopathology in dementia. *Neurology* 44 2308–2314. 10.1212/wnl.44.12.2308 7991117

[B27] Di Tella S. Pagliari C. Blasi V. Mendozzi L. Rovaris M. Baglio F. (2019). Integrated telerehabilitation approach in multiple sclerosis: a systematic review and meta-analysis. *J. Telemed. Telecare* 26 385–399. 10.1177/1357633x19850381 31132909

[B28] Dodakian L. Mckenzie A. L. Le V. See J. Pearson-Fuhrhop K. Burke Quinlan E. (2017). A home-based telerehabilitation program for patients with stroke. *Neurorehabil. Neural Repair* 31 923–933. 10.1177/1545968317733818 29072556 PMC5734923

[B29] Espay A. J. Bonato P. Nahab F. B. Maetzler W. Dean J. M. Klucken J. (2016). Technology in Parkinson’s disease: challenges and opportunities. *Mov. Disord.* 31 1272–1282. 27125836 10.1002/mds.26642 PMC5014594

[B30] Fetta J. Starkweather A. Gill J. M. (2017). Computer-based cognitive rehabilitation interventions for traumatic brain injury: a critical review of the literature. *J. Neurosci. Nurs.* 49 235–240. 10.1097/jnn.0000000000000298 28661947 PMC5510482

[B31] Folstein M. F. Folstein S. E. Mchugh P. R. (1975). “Mini-mental state”: a practical method for grading the cognitive state of patients for the clinician. *J. Psychiatr. Res.* 12 189–198. 1202204 10.1016/0022-3956(75)90026-6

[B32] Forducey P. G. Ruwe W. D. Dawson S. J. Scheideman-Miller C. Mcdonald N. B. Hantla M. R. (2003). Using telerehabilitation to promote TBI recovery and transfer of knowledge. *NeuroRehabilitation* 18 103–111. 10.3233/nre-2003-18203 12867673

[B33] Frasson P. Ghiretti R. Catricalà E. Pomati S. Marcone A. Parisi L. (2011). Free and cued selective reminding test: an Italian normative study. *Neurol. Sci.* 32 1057–1062. 10.1007/s10072-011-0607-3 21594655

[B34] Fratiglioni L. De Ronchi D. Aguero-Torres H. (1999). Worldwide prevalence and incidence of dementia. *Drugs Aging* 15 365–375. 10.2165/00002512-199915050-00004 10600044

[B35] Giovagnoli A. R. Del Pesce M. Mascheroni S. Simoncelli M. Laiacona M. Capitani E. (1996). Trail making test: normative values from 287 normal adult controls. *Ital. J. Neurol. Sci.* 17 305–309. 10.1007/bf01997792 8915764

[B36] Gooding A. L. Choi J. Fiszdon J. M. Wilkins K. Kirwin P. D. Van Dyck C. H. (2016). Comparing three methods of computerised cognitive training for older adults with subclinical cognitive decline. *Neuropsychol. Rehabil.* 26 810–821. 10.1080/09602011.2015.1118389 26674122

[B37] Hailey D. Roine R. Ohinmaa A. Dennett L. (2011). Evidence of benefit from telerehabilitation in routine care: a systematic review. *J. Telemed. Telecare* 17 281–287. 10.1258/jtt.2011.101208 21844172

[B38] Hong Y. J. Jang E. H. Hwang J. Roh J. H. Lee J. H. (2015). The efficacy of cognitive intervention programs for mild cognitive impairment: a systematic review. *Curr. Alzheimer Res.* 12 527–542. 10.2174/1567205012666150530201636 26027815

[B39] Isernia S. Pagliari C. Jonsdottir J. Castiglioni C. Gindri P. Gramigna C. (2019). Efficiency and patient-reported outcome measures from clinic to home: the human empowerment aging and disability program for digital-health rehabilitation. *Front. Neurol.* 10:1206. 10.3389/fneur.2019.01206 31824398 PMC6882300

[B40] Janoutova J. Sery O. Hosak L. Janout V. (2015). Is mild cognitive impairment a precursor of Alzheimer’s disease? Short review. *Cent. Eur. J. Public Health* 23 365–367. 10.21101/cejph.a4414 26841152

[B41] Jelcic N. Agostini M. Meneghello F. Busse C. Parise S. Galano A. (2014). Feasibility and efficacy of cognitive telerehabilitation in early Alzheimer’s disease: a pilot study. *Clin. Interv. Aging* 9 1605–1611. 10.2147/cia.s68145 25284993 PMC4181448

[B42] Kairy D. Lehoux P. Vincent C. Visintin M. (2009). A systematic review of clinical outcomes, clinical process, healthcare utilization and costs associated with telerehabilitation. *Disabil. Rehabil.* 31 427–447. 10.1080/09638280802062553 18720118

[B43] Katz S. (1983). Assessing self-maintenance: activities of daily living, mobility, and instrumental activities of daily living. *J. Am. Geriatr. Soc.* 31 721–727. 10.1111/j.1532-5415.1983.tb03391.x 6418786

[B44] Kidd P. M. (2008). Alzheimer’s disease, amnestic mild cognitive impairment, and age-associated memory impairment: current understanding and progress toward integrative prevention. *Altern. Med. Rev.* 13 85–115. 18590347

[B45] Lampit A. Hallock H. Moss R. Kwok S. Rosser M. Lukjanenko M. (2014). The Timecourse of global cognitive gains from supervised computer-assisted cognitive training: a randomised, active-controlled trial in elderly with multiple dementia risk factors can computerized cognitive training reverse the diagnosis of HIV-associated neurocognitive disorder? A research protocol. *J. Prev. Alzheimers Dis.* 1 33–39. 29261218 10.14283/jpad.2014.18

[B46] Lawton M. Brody E. (1988). Instrumental activities of daily living (IADL) scale-self-rated version. *Psychopharmacol. Bull.* 24 789–791. 3249786

[B47] Lezak M. Howieson D. Bigler E. Tranel D. (2012). *Neuropsychological Assessment*, 5th Edn Oxford: Oxford University Press.

[B48] Li B. Y. Wang Y. Tang H. D. Chen S. D. (2017). The role of cognitive activity in cognition protection: from Bedside to Bench. *Transl. Neurodegener.* 6:7. 10.1186/s40035-017-0078-4 PMC5371186 28360996

[B49] Li H. Li J. Li N. Li B. Wang P. Zhou T. (2011). Cognitive intervention for persons with mild cognitive impairment: a meta-analysis. *Ageing Res. Rev.* 10 285–296. 10.1016/j.arr.2010.11.003 21130185

[B50] Li R. Zhu X. Yin S. Niu Y. Zheng Z. Huang X. (2014). Multimodal intervention in older adults improves resting-state functional connectivity between the medial prefrontal cortex and medial temporal lobe. *Front. Aging Neurosci.* 6:39. 10.3389/fnagi.2014.00039 24653698 PMC3948107

[B51] Liao Y. Y. Chen I. H. Lin Y. J. Chen Y. Hsu W. C. (2019). Effects of virtual reality-based physical and cognitive training on executive function and dual-task gait performance in older adults with mild cognitive impairment: a randomized control trial. *Front. Aging Neurosci.* 11:162. 10.3389/fnagi.2019.00162 31379553 PMC6646677

[B52] Linden M. Hawley C. Blackwood B. Evans J. Anderson V. O’rourke C. (2016). Technological aids for the rehabilitation of memory and executive functioning in children and adolescents with acquired brain injury. *Cochrane Database Syst. Rev.* 7:CD011020. 10.1002/14651858.CD011020.pub2 PMC6457968 27364851

[B53] Livingston G. Sommerlad A. Orgeta V. Costafreda S. G. Huntley J. Ames D. (2017). Dementia prevention, intervention, and care. *Lancet* 390 2673–2734. 28735855 10.1016/S0140-6736(17)31363-6

[B54] Mashima P. A. Doarn C. R. (2008). Overview of telehealth activities in speech-language pathology. *Telemed. J. E Health* 14 1101–1117. 10.1089/tmj.2008.0080 19119834

[B55] Matamala-Gomez M. Maisto M. Montana J. I. Mavrodiev P. A. Baglio F. Rossetto F. (2020). The role of engagement in teleneurorehabilitation: a systematic review. *Front. Neurol.* 11:354. 10.3389/fneur.2020.00354 32435227 PMC7218051

[B56] McCue M. Fairman A. Pramuka M. (2010). Enhancing quality of life through telerehabilitation. *Phys. Med. Rehabil. Clin. N. Am.* 21 195–205. 10.1016/j.pmr.2009.07.005 19951786

[B57] Medalia A. Freilich B. (2008). The neuropsychological educational approach to cognitive remediation (NEAR) model: practice principles and outcome studies. *Am. J. Psychiatr. Rehabil.* 11 123–143. 10.1080/15487760801963660

[B58] Miceli G. Laudanna A. Burani C. Capasso R. (1994). *Batteria per l’Analisi dei Deficit Afasici (BADA), CEPSAG.* Rome: Universita Cattolica del Sacro Cuore.

[B59] Moreno A. Wall K. J. Thangavelu K. Craven L. Ward E. Dissanayaka N. N. (2019). A systematic review of the use of virtual reality and its effects on cognition in individuals with neurocognitive disorders. *Alzheimers Dement.* 5 834–850. 10.1016/j.trci.2019.09.016 31799368 PMC6881602

[B60] Morris J. C. (1997). Clinical dementia rating: a reliable and valid diagnostic and staging measure for dementia of the Alzheimer type. *Int. Psychogeriatr.* 9(Suppl. 1), 173–176; discussion 177–178. 9447441 10.1017/s1041610297004870

[B61] Moyle W. (2019). The promise of technology in the future of dementia care. *Nat. Rev. Neurol.* 15 353–359. 10.1038/s41582-019-0188-y 31073242

[B62] Novelli G. Papagno C. Capitani E. Laiacona M. (1986). Tre test clinici di ricerca e produzione lessicale. Taratura su sogetti normali. *Arch. Psicol. Neurol. Psichiatr.* 47 477–506.

[B63] Nucci M. Mapelli D. Mondini S. (2012). Cognitive reserve index questionnaire (CRIq): a new instrument for measuring cognitive reserve. *Aging Clin. Exp. Res.* 24 218–226. 21691143 10.3275/7800

[B64] Peres S. C. Pham T. Phillips R. (2013). “Validation of the system usability scale (SUS) SUS in the wild,” in *Proceedings of the Human Factors and Ergonomics Society Annual Meeting* (Los Angeles, CA: SAGE Publications), 192–196. 10.1177/1541931213571043

[B65] Peretti A. Amenta F. Tayebati S. K. Nittari G. Mahdi S. S. (2017). Telerehabilitation: review of the state-of-the-art and areas of application. *JMIR Rehabil. Assist. Technol.* 4:e7. 10.2196/rehab.7511 28733271 PMC5544892

[B66] Petersen R. C. (2004). Mild cognitive impairment as a diagnostic entity. *J. Intern. Med.* 256 183–194. 10.1111/j.1365-2796.2004.01388.x 15324362

[B67] Petersen R. C. (2011). Clinical practice. Mild cognitive impairment. *N. Engl. J. Med.* 364 2227–2234. 21651394 10.1056/NEJMcp0910237

[B68] Petersen R. C. Caracciolo B. Brayne C. Gauthier S. Jelic V. Fratiglioni L. (2014). Mild cognitive impairment: a concept in evolution. *J. Intern. Med.* 275 214–228. 24605806 10.1111/joim.12190 PMC3967548

[B69] Petersen R. C. Smith G. E. Waring S. C. Ivnik R. J. Tangalos E. G. Kokmen E. (1999). Mild cognitive impairment: clinical characterization and outcome. *Arch. Neurol.* 56 303–308. 10.1001/archneur.56.3.303 10190820

[B70] Pitt R. Theodoros D. Hill A. J. Russell T. (2019). The impact of the telerehabilitation group aphasia intervention and networking programme on communication, participation, and quality of life in people with aphasia. *Int. J. Speech Lang. Pathol.* 21 513–523. 10.1080/17549507.2018.1488990 30200788

[B71] Poon P. Hui E. Dai D. Kwok T. Woo J. (2005). Cognitive intervention for community-dwelling older persons with memory problems: telemedicine versus face-to-face treatment. *Int. J. Geriatr. Psychiatry* 20 285–286. 10.1002/gps.1282 15717335

[B72] Proschan M. A. Follmann D. A. (1995). Multiple comparisons with control in a single experiment versus separate experiments: why do we feel differently? *Am. Stat.* 49 144–149. 10.2307/2684628

[B73] R Core Team (2013). *R: A Language and Environment for Statistical Computing.* Vienna: R Foundation for Statistical Computing.

[B74] Realdon O. Rossetto F. Nalin M. Baroni I. Cabinio M. Fioravanti R. (2016). Technology-enhanced multi-domain at home continuum of care program with respect to usual care for people with cognitive impairment: the ability-TelerehABILITation study protocol for a randomized controlled trial. *BMC Psychiatry* 16:425. 10.1186/s12888-016-1132-y 27887597 PMC5123349

[B75] Rosen M. J. (2004). Telerehabilitation. *Telemed. J. E Health* 10 115–117. 15319039 10.1089/tmj.2004.10.115

[B76] Sherman D. S. Mauser J. Nuno M. Sherzai D. (2017). The efficacy of cognitive intervention in mild cognitive impairment (MCI): a meta-analysis of outcomes on neuropsychological measures. *Neuropsychol. Rev.* 27 440–484. 10.1007/s11065-017-9363-3 29282641 PMC5754430

[B77] Shulman K. I. Pushkar Gold D. Cohen C. A. Zucchero C. A. (1993). Clock-drawing and dementia in the community: a longitudinal study. *Int. J. Geriatr. Psychiatry* 8 487–496. 10.1002/gps.930080606

[B78] Siciliano M. Chiorri C. Battini V. Sant’elia V. Altieri M. Trojano L. (2019). Regression-based normative data and equivalent scores for Trail Making Test (TMT): an updated Italian normative study. *Neurol. Sci.* 40 469–477. 10.1007/s10072-018-3673-y 30535956

[B79] Solana J. Cáceres C. García-Molina A. Opisso E. Roig T. Tormos J. M. (2015). Improving brain injury cognitive rehabilitation by personalized telerehabilitation services: guttmann neuropersonal trainer. *IEEE J. Biomed. Health Inform.* 19 124–131. 10.1109/jbhi.2014.2354537 25204001

[B80] Sunderland A. Watts K. Baddeley A. D. Harris J. E. (1986). Subjective memory assessment and test performance in elderly adults. *J. Gerontol.* 41 376–384. 10.1093/geronj/41.3.376 3700988

[B81] Topol E. (2019). *The Topol Review: Preparing the Healthcare Workforce to Deliver the Digital Future.* London: Health Education England.

[B82] Tuena C. Pedroli E. Trimarchi P. D. Gallucci A. Chiappini M. Goulene K. (2020). Usability issues of clinical and research applications of virtual reality in older people: a systematic review. *Front. Hum. Neurosci.* 14:93. 10.3389/fnhum.2020.00093 32322194 PMC7156831

[B83] Vance D. E. Fazeli P. L. Azuero A. Wadley V. G. Jensen M. Raper J. L. (2018). Can computerized cognitive training reverse the diagnosis of HIV-associated neurocognitive disorder? A research protocol. *Res. Nurs. Health* 41 11–18. 10.1002/nur.21841 29266286 PMC5780199

[B84] Vannini P. Amariglio R. Hanseeuw B. Johnson K. A. Mclaren D. G. Chhatwal J. (2017). Memory self-awareness in the preclinical and prodromal stages of Alzheimer’s disease. *Neuropsychologia* 99 343–349. 10.1016/j.neuropsychologia.2017.04.002 28385579 PMC5473166

[B85] Vermeij A. Claassen J. A. Dautzenberg P. L. Kessels R. P. (2016). Transfer and maintenance effects of online working-memory training in normal ageing and mild cognitive impairment. *Neuropsychol. Rehabil.* 26 783–809. 10.1080/09602011.2015.1048694 26010573

[B86] Yao S. Liu Y. Zheng X. Zhang Y. Cui S. Tang C. (2020). Do nonpharmacological interventions prevent cognitive decline? a systematic review and meta-analysis. *Transl. Psychiatry* 10:19. 10.1038/s41398-020-0690-4 PMC7026127 32066716

[B87] Yesavage J. A. Brink T. L. Rose T. L. Lum O. Huang V. Adey M. (1982). Development and validation of a geriatric depression screening scale: a preliminary report. *J. Psychiatr. Res.* 17 37–49. 10.1016/0022-3956(82)90033-4 7183759

[B88] Zampolini M. Todeschini E. Bernabeu Guitart M. Hermens H. Ilsbroukx S. Macellari V. (2008). Tele-rehabilitation: present and future. *Ann. Ist. Super. Sanita* 44 125–134. 18660562

